# Effects of Arbuscular Mycorrhizal Fungi-Colonized *Populus alba* × *P. berolinensis* Seedlings on the Microbial and Metabolic Status of Gypsy Moth Larvae

**DOI:** 10.3390/insects13111002

**Published:** 2022-10-31

**Authors:** Mingtao Tan, Yaning Li, Jinsheng Xu, Shanchun Yan, Dun Jiang

**Affiliations:** 1 School of Forestry, Northeast Forestry University, Harbin 150040, China; 2 Key Laboratory of Sustainable Forest Ecosystem Management-Ministry of Education, Northeast Forestry University, Harbin 150040, China

**Keywords:** arbuscular mycorrhizal fungi, *Lymantira dispar*, gut microbiota, metabolism

## Abstract

**Simple Summary:**

Arbuscular mycorrhizal fungi can be used as a potential alternative to chemical pesticides for pest control. In this study, two mycorrhizal fungi (*Glomus mossae* and *Glomus intraradices*), *P. alba × P. berolinensis* seedlings, and gypsy moth larvae were successfully used to investigate the basis of mycorrhiza-induced resistance/susceptibility at the larval microbial and metabolic levels. The disadaptation of gypsy moth larvae to the leaves of GM-colonized seedlings, such as the gut microbial dysfunction and fat body metabolic disorder, is the main reason for GM-induced insect resistance. However, the improvement of gut environment and fat body metabolism in gypsy moth larvae results in the GI-induced insect susceptibility.

**Abstract:**

Arbuscular mycorrhizal fungi (AMF) are considered as important biological factors that can affect insect resistance of plants. Herein, we used AMF-poplar seedlings that could either increase or decrease the resistance to gypsy moth larvae, to elucidate the mechanism of mycorrhizal-induced insect resistance/susceptibility at the larval microbial and metabolic levels. Our results found that larval plant consumption and growth were significantly inhibited in the *Glomus mossae* (GM)-colonized seedlings, whereas they were enhanced in the *Glomus intraradices* (GI)-colonized seedlings. GM inoculation reduced the beneficial bacteria abundance in the larval gut and inhibited the detoxification and metabolic functions of gut microbiota. However, GI inoculation improved the larval gut environment by decreasing the pathogenic bacteria and activating specific metabolic pathways. Furthermore, GM inoculation triggers a metabolic disorder in the larval fat body, accompanied by the suppression of detoxification and energy production pathways. The levels of differentially accumulated metabolites related to amino acid synthesis and metabolism and exogenous toxin metabolism pathways were significantly increased in the GI group. Taken together, the disadaptation of gypsy moth larvae to leaves of GM-colonized seedlings led to the GM-induced insect resistance in poplar, and to the GI-induced insect susceptibility involved in the improvement of larval gut environment and fat body energy metabolism.

## 1. Introduction

Arbuscular mycorrhizal fungi (AMF), belonging to the Glomeromycota phylum, are widely distributed microorganisms associated with the plant roots. During the long-term co-evolution process, AMF have established symbiotic relationships with more than 80% of vascular plant species on the Earth [1]. Generally, the host plant provides carbohydrates synthesized by photosynthesis to the AMF as carbon sources. Meanwhile, AMF colonization improves the absorption and utilization of nitrogen, phosphorus, and other nutrients in the soil by plants and significantly promotes the plants’ growth [2,3]. In addition, AMF can also improve plant resistance to various environmental stresses, including salt stress, heavy metal stress, drought stress, and temperature stress, by promoting the physical and chemical properties of soil [4,5,6,7,8].

As an important part of the terrestrial ecosystem, phytophagous insects easily form a complex interaction with the AMF and plants. Studies have shown that AMF, as a biological stimulator, can improve the plant resistance to phytophagous insects and weaken the growth of these insects by triggering the defense response in plants [9]. Formenti et al. (2019) showed that inoculation with the *Rhizophagus irregularis* promoted jasmonic acid synthesis in tomatoes and inhibited the feeding of *Spodoptera Mauritia* [10]. Schoenherr et al. (2019) also reported that potatoes inoculated with *R. irregularis* could significantly inhibit the growth of *Trichoplusia ni* [11]. Further, AMF inoculation has been shown to significantly increase the phenolic content in *Triticum aestivum* and inhibit the relative growth rate of *Helicoverpa punctigera* [12]. A hypothesis, the mycorrhiza-induced resistance (MIR) hypothesis, has been proposed to explain these biological phenomena [13]. The MIR refers to the induction of plant resistance to phytophagous insects by activating the defense signaling pathway following the establishment of a symbiotic relationship between AMF and plants. Due to the existence of MIR, AMF can be used as a potential alternative to chemical pesticides for pest control in agroforestry production.

Interestingly, MIR is not induced in all the AMF–plant systems [14,15,16]. In recent years, few studies have shown that AMF increased the growth and development of insects after forming a symbiotic relationship with the plant. *Mamestra brasicae* gained weight and a shortened development duration after feeding on *Plantago lanceolata* colonized by *R. irregularis* [14]. In a similar study, Real-Santillan et al. (2019) reported that *Zea mays* inoculated with *Glomus* spp., *Acaulospora* spp., *Gigaspora* spp., and *Intraspora* spp. promoted the growth and development of *Spodoptera frugiperda* larvae [15]. These phenomena are collectively termed mycorrhizal-induced susceptibility (MIS). In addition, the influence of mycorrhiza on the growth and development of insects also has a neutral effect, which is called mycorrhizal-induced neutra (MIN). Minton et al. (2016) showed that *Solanum ptycanthum* and *S. dulcamara* inoculated with *R. irregularis* did not affect the growth and development of the phytophagous insect *Manduca sexta* [16]. Therefore, it should be noted that the effects of AMF colonization on plant resistance to insects vary in the field and are dependent on various factors.

Based on the importance of AMF in agriculture and forestry, a large number of studies have systematically explored the interaction between AMF-agricultural and forestry plants and phytophagous insects and have proposed different hypotheses related to describing plant resistance to the insects, such as MIR, MIS, and MIN. However, the studies available to date that decipher the mechanism of AMF affecting plant resistance to insects only focus on the physiological and cellular changes in the plants. The systematic investigation of AMF-induced plant resistance from the perspective of insects is still lacking. In our previous studies, we found that the inoculation with *Glomus mossae* (GM) improved the resistance to gypsy moth larvae in *Populus alba × P. berolinensis* seedlings, while inoculation with *Glomus intraradices* (GI) resulted in the opposite effect [17,18]. In the current study, leaves of *P. alba × P. berolinensis* inoculated with GM or GI were used to feed gypsy moth larvae, and the response of the gut microbiota and fat body metabolome in gypsy moth larvae to AMF was characterized. These findings will be beneficial in understanding how mycorrhiza-induced resistance/susceptibility is triggered from the insect perspective and further expand our knowledge of the AMF-plant-phytophagous-insect interaction.

## 2. Materials and Methods

### 2.1. Treatment of P. alba × P. berolinensis Seedlings

The flowerpots, turfy soil, sand, vermiculite, and the annual cuttings of *P. alba × P. berolinensis* seedling were purchased from Harbin Flower Market (Harbin, China). Turfy soil, sand, and vermiculite were carefully mixed in a 3:1:1 volume ratio at the nursery of Northeast Forestry University and then sterilized in an autoclave at 121 °C for 2 h. The cuttings of *P. alba* × *P. berolinensis* seedlings and flowerpots were sterilized with 2% KMnO_4_ solution for 30 min. The flowerpots with a diameter of 250 mm and a height of 230 mm were divided into 3 groups. Among them, the untreated group (denoted as CK) was only loaded with a 2 kg soil mixture. The other two groups were filled with a homogeneous mixture of 2 kg of sterilized soil and 20 g of GM (denoted as GM) or GI (denoted as GI). Both GM (BGC XJ08A) and GI (BGC BJ09) microbial inocula were purchased from the Gansu Academy of Agricultural Sciences, China. This mycorrhizal inoculum was composed of spores, mycelium, root segments, and sand, and the number of spores provided in 1 g of mycorrhizal inoculum was about 15. Subsequently, one seedling was planted in each flowerpot at the end of April 2020, and a total of 150 seedlings were planted in each group. After the 30th, 60th, and 90th days of AMF infection, root samples of the poplar seedlings from three groups were harvested and were then stained with 0.008% trypan blue as previously described [19]. The root colonization by AMF was observed by a low-power compound microscope, and the presence of spores or arbuscules in the root segment was considered as a successful colonization. There were 4 replicates in CK, GI, or GM groups, and at least 50 root segments were observed in each replicate. The root colonization rate was calculated according to the following formula: Root colonization (%) = 100 × Number of infected root segments/Total number of root segments observed. Data were presented as percentage AM fungus root colonization and analyzed using the One-way ANOVA followed by LSD multiple comparisons at the 0.05 level.

### 2.2. Insect Rearing

The egg masses of Asian gypsy moth were obtained from the campus of Northeast Forestry University (Harbin, China) in March 2020. All the egg masses were provided by the Insect Chemical Ecology Lab (college of forestry, Northeast Forestry University). At the beginning of August, eggs of the gypsy moth were sterilized with 10% formaldehyde solution for 1 h and incubated for hatching at 25 °C with a 16 L: 8D photoperiod and 60 ± 1% relative humidity. After hatching, the gypsy moth larvae were fed an artificial diet in the same environment until the second instar. The artificial diet was purchased from the Chinese Academy of Forestry Sciences (Beijing, China). The newly molted 2nd-instar larvae were divided into 3 groups, which were fed with young leaves of the GM group, GI group, and CK group. The larvae were kept in a plastic box. Each plastic box contained five larvae and 10 leaves. Each group was designed with 4 replicates, and each replicate consisted of 25 larvae. During the experiment, the leaves were changed every two days. Thirty gypsy moth larvae were randomly selected from 4 replicates of each group, and their body weight, plant consumption, and developmental period were recorded. Subsequently, the plant consumption, growth rate, and relative growth rate of gypsy moth larvae were calculated according to the following formula: Plant Consumption = fresh leaf weight—residual leaf weight; Growth Rate = (final weight-initial weight)/T; Relative Growth Rate = (final weight-initial weight)/(initial weight × T) [20]. Here, fresh leaf weight and residual leaf weight refer to the leaf weight before feeding and the leaf weight after feeding for 48 h, respectively; final weight and initial weight, respectively, indicate the weight of larvae at the end and beginning of an instar; T represents the number of days between final weight and initial weight. SPSS 26.0 software was used to analyze the data by one-way ANOVA, and the LSD (least significant method) was used to test the significant difference between the treatment group and the control group with a *p*-value < 0.05.

### 2.3. Gut Microbiota Analysis

The newly molted gypsy moth larvae at the 5th instar were anesthetized on ice, and their gut tissues were dissected. The gut tissue of 5 larvae was taken as one repeat. Similarly, 4 repeats were set for both the control and treatment groups. Gut flora DNA of gypsy moth larvae was extracted using the EZN.A^®^ Stool kit. After qualitative detection on 0.8% agarose gel electrophoresis, all DNA samples were sent to the LianChuan Biotechnology Co., Ltd. (China, Hangzhou) for gut microbiota analysis. The 5′ ends of the primers were tagged with specific barcodes per sample and sequencing universal primers. PCR amplification was performed in a total volume of 25 μL of reaction mixture containing 25 ng of template DNA, 12.5 μL of PCR Premix, 2.5 μL of each primer, and PCR-grade water to adjust the volume. After confirming with 2% agarose gel electrophoresis, the PCR products were purified by AMPure XT beads (Beckman Coulter Genomics, Danvers, MA, USA) and quantified by Qubit (Invitrogen, CA, USA). The amplicon pools were prepared for sequencing and the size and quantity of the amplicon library were assessed on an Agilent 2100 Bioanalyzer (Agilent, PA, USA) and with the Library Quantification Kit for Illumina (Kapa Biosciences, Woburn, MA, USA), respectively. The libraries were sequenced on the NovaSeq PE250 platform. Samples were sequenced on an Illumina NovaSeq platform according to the manufacturer’s recommendations. Paired-end reads were assigned to samples based on their unique barcode and truncated by cutting off the barcode and primer sequence. Paired-end reads were merged using FLASH. Quality filtering on the raw reads were performed under specific filtering conditions to obtain the high-quality clean tags according to the fqtrim (v0.94). Chimeric sequences were filtered using Vsearch software (v2.3.4). After dereplication using DADA2, the feature table and feature sequence were obtained. The alpha diversity (Shannon and Simpson index) of gut microorganisms was calculated using QIIME2, and the beta diversity was calculated using unweighted principal coordinate analysis (PCoA) and principal component analysis (PCA). Blast was used for sequence alignment, and the feature sequences were annotated with the SILVA database for each representative sequence. Linear discriminant analysis (LDA) and the effect size (LEFSe) bifurcation diagram were used to identify the microbial groups with different relative abundance between control and treatment groups. PICRUSt2 (phylogenetic investigation of communities by reconstruction of unobserved states 2) was used to predict gut microbial function. Further, an independent sample *t*-test was used to evaluate the difference in the relative abundance of gut microorganisms with the same functions between CK and GM groups or CK and GI groups.

### 2.4. Metabolomic Analysis of Larval Fat Body

The newly molted gypsy moth larvae at the 5th instar stage were dissected on ice to obtain the fat body. The fat body collected from 5 larvae was regarded as one repeat. Similarly, each group was set with four repeats. Samples were sent to the China LianChuan Biotechnology Co., Ltd. for nontargeted metabolomics analysis. In brief, a 100 mg tissue sample was weighed and ground in the liquid nitrogen. Subsequently, 150 μL of 50% methanol was added, mixed evenly by shaking, and incubated at room temperature for 10 min. The crude extract was placed in a refrigerator at −20 °C overnight for protein precipitation. The crude was centrifuged at 4000 *g* to obtain the supernatant, which was used further as the metabolite extract. Isopropanol, acetonitrile, and water (2:1:1) were used to dilute the metabolite extract. The metabolite extracts were analyzed using a high-resolution mass spectrometer TripleTOF 5600plus (SCIEX, Warrington, UK), and chromatographic separation was performed using an ultra-performance liquid chromatography (UPLC) system (SCIEX, Warrington, UK). The LC-MS data were processed by the XCMS software. The original data file was converted to mzXML format and then processed using the CAMERA and metaX toolbox tools. The identified metabolites were annotated using KEGG databases. Partial least squares–Discriminant analysis (PLS–DA) was used to study the relationship between metabolite expression in CK, GM, and GI groups. Differentially accumulated metabolites (DAMs) were identified with a fold change (FC) > 2 and *p* values < 0.05. Further, pathway analysis of differentially accumulated metabolites was performed using MetaboAnalysis (https://www.metaboanalyst.ca/; accessed on 8 May 2022).

## 3. Results

### 3.1. Root Colonization by AMF

A mutualistic association between the commercial mycorrhizal inoculum *G. intraradices* or *G. mosseae* and the *P. alba × P. berolinensis* seedlings was successfully established (Table S1). The root colonization rates by *G. intraradices* or *G. mosseae* increased with the extension of treatment time, and the highest root colonization rates in GM and GI groups were 72.46% and 82.3%, respectively. In each sampling period, the root colonization rates of the GM group were significantly lower than those of the GI group.

### 3.2. Analysis of Growth and Development Indexes of Gypsy Moth Larvae

**Figure 1:**
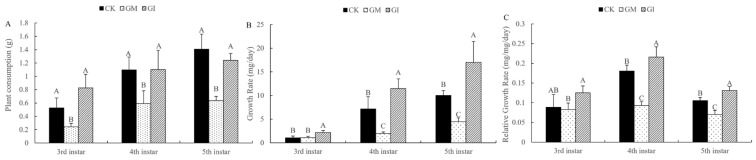
The plant consumption (**A**), growth rate (**B**), and relative growth rate (**C**) of the 3rd-, 4th-, and 5th-instar gypsy moth larvae fed on the leaves of GM, GI, or nonmycorrhizal-colonized poplar plants. Different capital letters indicate significant differences among groups (*p* < 0.05).

To evaluate the effect of GM- or GI-colonized-populus on the growth and development of gypsy moth, the plant consumption, growth rate, and relative growth rate of gypsy moth larvae were measured. The results showed that the plant consumption of gypsy moth larvae at the 3rd, 4th, and 5th instar in the GM treatment group was significantly lower than that in the untreated group and GI treatment group, while the larval plant consumption in the GI group was not significantly different from that in the CK group (Figure 1A). As shown in Figure 1B, the growth rates of 4th- and 5th-instar larvae of gypsy moth in the GM inoculation group were significantly lower than those in the CK group, while the growth rates of 3rd–5th-instar larvae in the GI treatment group were higher as compared to the CK group. After GM and GI treatments, the relative growth rate of larvae was consistent with the growth rate. The relative growth rate of 4th- and 5th-instar larvae was significantly lower and higher in the GM and GI groups as compared to the untreated group, respectively (Figure 1C).

### 3.3. Diversity Analysis of Gut Microbial Community

Based on the 16S rDNA sequencing, about 888,230 high-quality sequences were obtained from 12 samples of CK, GM, and GI groups. After classification, 598 operational taxonomic units (OTU) were identified. The Venn diagram showed that 65 OTUs were common among the CK, GM, and GI groups, 23 OTUs were common between CK and GM groups, 25 OTUs were common between CK and GI groups, and 18 OTUs were common between GM and GI groups. In addition, 224, 145, and 98 unique OTUs were identified in the CK, GM, and GI groups, respectively. The Venn diagram showed that the OTU composition of gut microflora of gypsy moth larvae was significantly changed by feeding on *P. alba × P. berolinensis* inoculated with GM or GI (Figure S1).

**Figure 2:**
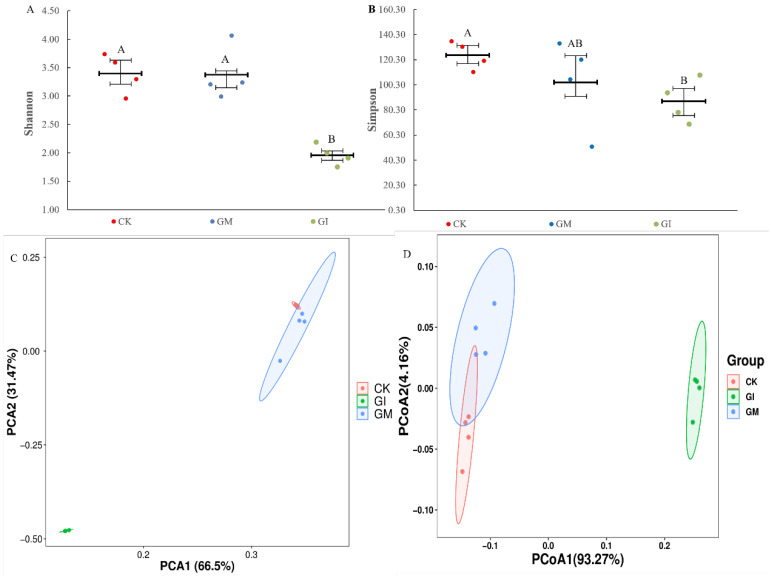
The gut microbial diversity in the gypsy moth larvae at the 5th instar after rearing on the leaves of GM, GI, or n nonmycorrhizal-colonized plants. (**A**): Shannon index. (**B**): Simpson index. (**C**): Principal component analysis (PCA). (**D**): PCoA plot of the gut microbiota structures based on the unweighted UniFrac analysis. Different capital letters indicate significant differences between treatment groups or control group (*p* < 0.05).

The total sequences obtained by 16S rDNA sequencing were aligned, and the richness and species differences within the gut microbial community of gypsy moth larvae were evaluated by comparing α and β diversity. The results showed that compared to the untreated group, the Shannon and Simpson indexes of gut microflora in the GM treatment group had no significant change, whereas these indexes were decreased markedly in the GI group (Figure 2A,B). PCA showed that the gut microflora in CK, GM, and GI groups were well isolated, indicating that the similarity coefficient of species community among the three groups was low (*p* < 0.05, Figure 2C). At the same time, PCoA analysis also revealed a significant difference in the gut microbiota composition among the three groups (*p* = 0.001, Figure 2D).

### 3.4. Differential Gut Microflora Analysis

A total of 18 bacterial phyla were identified in the CK, GM, and GI groups. LefSe analysis showed that the abundance of Firmicutes in the untreated group was higher than that in the GM group, while the abundance of Cyanobacteria in the GM group was higher than that in the CK group (Figure S2A,B). The relative abundance of four phyla was significantly different between the untreated and GI groups. The untreated group was characterized by a higher abundance of Proteobacteria, Firmicutes, and Bacteroidetes, while the GI group was characterized by a higher abundance of Cyanobacteria (Figure S3A,B). At the genus level, 250 bacterial genera were identified in the CK, GM, and GI groups (Table S2). There were significant differences in the relative abundance of 15 genera between the CK and GM groups. The abundance of 8 bacterial genera increased significantly in the CK group, whereas the abundance of 7 bacterial genera increased markedly in the GM group (Figure S2A,B, Table S3). There were noticeable differences in the relative abundance of 28 genera between CK and GI groups. Among them, the abundance of 19 bacterial genera was found to be increased in the CK group, whereas the abundance of 9 bacterial genera was increased in the GI group (Figure S3A,B, Table S4)

### 3.5. Analysis of Gut Microbial Function

The KEGG database was used to evaluate the functional alterations in the gut microbial community among control/treatment groups, and an independent sample *t*-test was used for significance analysis. As shown in Figure S4A, glycan biosynthesis and metabolism and digestive system pathways were significantly down-regulated, whereas cardiovascular diseases, amino acid metabolism, metabolism of other amino acids, the endocrine system, neurodegenerative diseases, cancers, transport and catabolism, the circulatory system, and metabolism pathways were up-regulated in the GM group as compared to the CK group. The expression levels of the biosynthesis of other secondary metals, cellular processes and signaling, lipid metabolism, transport and catabolism, transcription, membrane transport, and metabolism pathways in the GI treatment group were significantly lower than those in the CK group, while cell growth and death, genetic information processing, metabolic diseases, metabolism of other amino acids, metabolism of terpenoids and polyketides, the immune system, the excretory system, the digestive system, folding, sorting and degradation, metabolism of cofactors and vitamins, enzyme families, immune system diseases, and energy metabolism pathways were markedly higher than those in the CK group (Figure S4B).

### 3.6. Metabonomics Analysis

**Figure 3:**
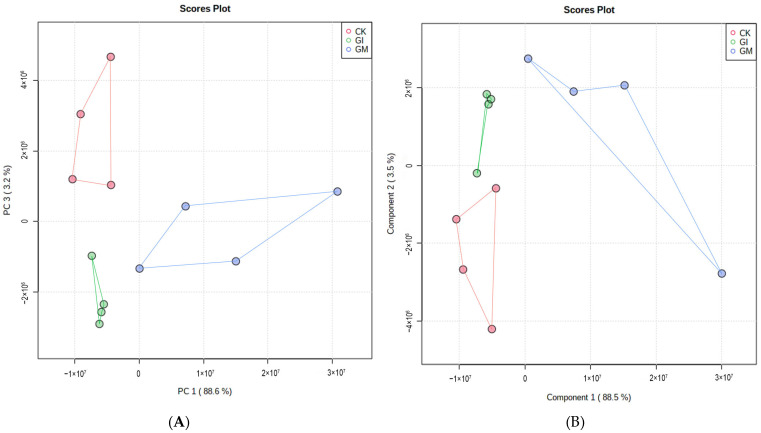
The principal components analysis (PCA, (**A**)) and supervised partial least squared discriminant analysis (PLS–DA, (**B**)) of metabolite profiles in fat body of gypsy moth larvae.

**Figure 4:**
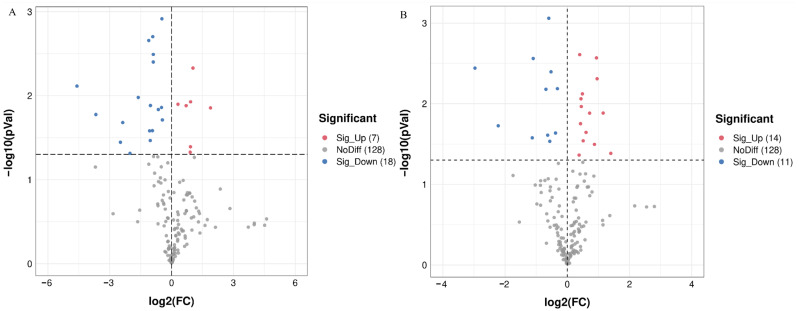
Volcano plots showing the differentially expressed metabolites among GM and CK groups (**A**) or among GI and CK groups (**B**). All metabolites presented in volcano plots were annotated through the KEGG database. Sig_Up, significantly up-regulated among control and treatment groups (*p* < 0.05). Sig_Down, significantly down-regulated among control and treatment groups (*p* < 0.05). NoDiff, no significant difference among control and treatment groups (*p* > 0.05).

**Figure 5:**
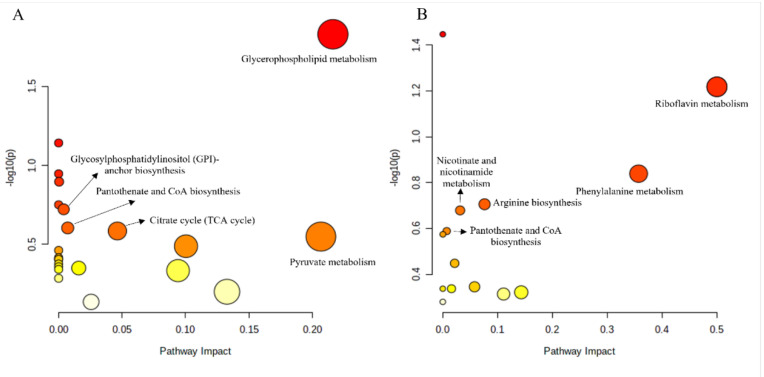
Pathway analysis of the differentially expressed metabolites between CK group and GM group (**A**) or CK group and GI group (**B**). The *x*-axis (−log_10_ (*p*-value)) represents the pathway enrichment score and the *y*-axis represents the pathway impact score. Pathway impact score is proportional to the bubble size. The significance level of the pathways is denoted by the bubble color, ranging from highest (red) to lowest (white).

To compare the metabolic differences between treated and untreated groups, the metabolites isolated from the fat body of gypsy moth larvae were analyzed under the positive ion (ESI +) mode of UPLC-QTOF MS. A total of 20,687 target peaks were detected, of which 153 were annotated using KEGG. The PCA analysis showed that there was a good separation among the three groups. Principal components PC1 and PC2 accounted for 88.6% and 4.1% of the changes, respectively (Figure 3A). PLS–DA analysis further revealed considerable segregation between the CK, GM, and GI groups (Figure 3B). FC analysis followed by the t-test led to the identification of 25 DAMs in the GM and GI groups (Figure 4A,B). Compared to CK, the contents of 18 metabolites decreased significantly in the GM-treated group, while the contents of 7 metabolites increased (Table S5). For the GI-treated group, the contents of 14 metabolites were significantly reduced, while the contents of 11 metabolites were markedly increased compared to that of CK (Table S6). KEGG enrichment analysis combined with the pathway topological characteristics was used to evaluate the influence of DAMs on KEGG pathways. Post-GM treatment, glycerophospholipid metabolism, glycosylphosphatidylinositol (GPI)-anchor biosynthesis, pantothenate and CoA biosynthesis, the citrate cycle (TCA cycle), and pyruvate metabolism were found to be affected (Figure 5A). Further, riboflavin metabolism, phenylalanine metabolism, arginine biosynthesis, nicotinate and nicotinamide metabolism, and pantothenate and CoA biosynthesis pathways were significantly altered after GI treatment (Figure 5B).

## 4. Discussion

As proposed by the MIR hypothesis, the insect resistance of plants colonized with AMF is improved by increasing the content of toxic secondary metabolites or decreasing the palatability of plants. For example, Wang et al. (2020) found that AMF colonization significantly increased the phenolic content in wheat and reduced the number of wheat aphids [21]. It is generally believed that the “AMF-plant” symbiotic system has ecological specificity [22,23]. In some cases, this ecological specificity inhibits plant resistance and promotes the growth of harmful insects. In line with our previous studies, the present study revealed that the GM group induced the resistance of *P. alba × P. berolinensis* seedlings to gypsy moth, which was manifested by a significant decrease in larval plant consumption, growth rate, and relative growth rate, while GI reduced the resistance of *P. alba × P. berolinensis* seedlings to gypsy moth [17,18]. These results, together with the previous studies, demonstrated that specific AMF colonization could induce alterations (either increase or decrease) in the plant resistance to insects. Therefore, for the utilization of AMF in agriculture and forestry production, AMF-plant combinations concerning insect resistance should be selected carefully to avoid the positive effects of AMF colonization on the growth of herbivorous insects.

During the long-term evolution process, insects and their gut microbiota have established a mutually beneficial symbiotic relationship [24,25,26]. As one of the main factors to maintain homeostasis in host insects, the gut microbiota is regarded as an important index to measure the growth of host insects. In the present study, different gut microbiota at the genus level were identified between the untreated and treated groups by LefSe analysis, and subsequently, their functions were elucidated. The results showed that the relative abundance of 8 genera in the gypsy moth larvae gut in the GM group was decreased as compared to the untreated group. Of these, two beneficial bacteria (e.g., *Acinetobacter* and *Ralstonia*) involved in energy metabolism, detoxification metabolism, or improvement of the intestinal barrier were identified [27,28]. A decrease in these two genera in the GM group indicated that the digestion of food materials and the degradation ability of plant secondary substances were reduced in the gypsy moth larvae. Surprisingly, the abundance of some beneficial bacteria (*Staphylococcus* and *Klebsiella*) that can promote digestion and absorption in the host significantly increased in the GM group, suggesting that gypsy moth larvae try to compensate for the nutrition obtained from low-quality food with the help of gut microbiota [25,29]. The abundance of 20 genera in the GI group decreased significantly as compared to those in the untreated group. These significantly altered genera included some pathogenic bacteria (e.g., *Neisseria*, *Klebsiella,* and *Streptococcus*) [30,31,32]. These results indicate that GI treatment seems to improve the gut environment in gypsy moth larvae by reducing the abundance of pathogenic bacteria in the larval gut. In addition, beneficial bacteria (*Lachnospirnceae, Ruminococcaceae,* and *Lactobacillus*) associated with the nutritional metabolism and detoxification were also found to decrease in the GI group [33,34,35]. This may be related to the fact that the improved leaf quality in the GI group reduced the requirement of gut beneficial bacteria for gypsy moth larvae.

To further explore the functional diversity of gut microbiota in gypsy moth larvae between AMF-treated and untreated groups, PICRUSt2 function prediction and significance analysis were performed. We observed that glycan biosynthesis and metabolism and digestible system pathways in the GM group were down-regulated, indicating the inhibition of digestion, absorption, and energy metabolism in gut microbiota. In addition, the pathways related to cardiovascular diseases, neurodevelopmental diseases, and cancer were also up-regulated in the GM group, which suggests that the *P. alba × P. berolinensis* seedlings with GM colonization provide a better environment for the reproduction of pathogenic bacteria in the larval gut. However, the energy metabolism and detoxification pathways in the GI group were significantly up-regulated, including the metabolism of other amino acids, metabolism of terpenoids and polyketides, metabolism of cofactors and vitamins, and energy metabolism. It can be deduced that the ability of larvae to utilize nutrients and adapt to host plants increased in the GI treatment group. In addition, immune-related pathways such as immune system and enzyme families were significantly up-regulated in the GI group, which might be the reason behind the decrease in pathogenic bacteria in the GI group. Altogether, the decrease in gut beneficial bacteria and microbial function disorder is one of the main reasons for the growth retardation of gypsy moth larvae in the GM group, while the improved gut environment and the enhanced metabolic functions of gut flora are responsible for the increased larval growth in the GI group.

Metabolites, as important markers associated with various physiological and biochemical activities, are closely related to the growth and development of insects. The metabolic levels of gypsy moth larvae in the treated and untreated groups were analyzed by untargeted metabonomics. Our results revealed that 25 DAMs were identified in both GM and GI groups. In the comparative analysis of GM and CK groups, it was observed that several DAMs that are critical for growth, reproduction, and detoxification metabolism were significantly reduced in the GM group, such as uridine, 5, 6-dihydroxyindole-2-carboxylic acid, and penicillamine [36,37,38]. These results indicated that the metabolic disorder of gypsy moth larvae in the GM treatment group occurred, consistent with the growth retardation of gypsy moth larvae mentioned above. In addition, corticosterone (a metabolite), which can lead to a developmental disorder and a repressed immune system [39], was increased significantly in the fat body of GM larvae, highlighting the possible reason underlying the decreased adaptability of larvae to the leaves of *P. alba × P. berolinensis* colonized with GM. However, GI colonization significantly enhanced the metabolism in gypsy moth larvae. Some DAMs related to growth and development (e.g., (-)-Riboflavin, 3, 4-Dihydroxy-L-phenylalanine, and phenylalanine) showed a marked increase in the GI group, while other DAMs with toxic effects decreased significantly, such as Dodecanoic acid and Pyridin [40,41,42,43]. Among these, amino acids were the majorly up-regulated metabolites in the GI group. Previous studies have demonstrated that amino acids are positively correlated with the digestive level or growth of insects [44]. Therefore, the increase in amino acid content in the GI treatment group may account for the improvement of larval growth. Functional analysis of DAMs showed that the metabolites affected by GM were mainly involved in the energy metabolism and detoxification pathways, such as the citrate cycle (TCA cycle), pyruvate metabolism, and glycerophospholipid metabolism. Combined with the analysis of DAMs, it was found that the energy metabolism and detoxification ability disorder was another reason for the growth inhibition of gypsy moth larvae in the GM treatment group. DAMs increased by GI treatment were mainly involved in the amino acid synthesis and metabolism (e.g., phenylalanine metabolism and arginine biosynthesis), and exogenous toxin metabolism (e.g., nicotinate and nicotinamide metabolism, and pantothenate and CoA biosynthesis), elucidating that GI colonization further increased the ability of gypsy moth larvae to digest food and adapt to the chemical defense of plants.

In the present study, gut microbiota and fat body metabolism of gypsy moth larvae showed two completely opposite trends in response to GM or GI-colonized *Populus alba* × *P. berolinensis* seedlings. This is consistent with the growth of gypsy moth larvae. Our previous work found that GM colonization significantly activated the defense response of *Populus alba* × *P. berolinensis* seedlings, as evidenced by the several metabolites (e.g., coumarin, stachydrine, and artocarpin) associated with insect resistance increasing significantly. In contrast, GI colonization inhibited the flavonoid biosynthesis pathway at the transcriptional level, resulting in a significant decrease in the accumulation of flavonoid compounds in leaves, such as catechin and quercetin. It is not difficult to infer that the difference in leaf quality caused the discordance of larval gut microbiota and fat body metabolism in the GM and GI group. The current results, combined with our previous results, clearly show that in a mycorrhizal-plant–herbivore interaction, the secondary metabolic level of mycorrhizal plants regulates the physiological status of the herbivore and ultimately affects their growth and development.

## 5. Conclusions

Two mycorrhizal fungi (GM and GI), *P. alba × P. berolinensis* seedlings, and gypsy moth larvae were successfully used to investigate the basis of mycorrhiza-induced resistance/susceptibility at the physiological level in insects. The gut microbial function and fat body metabolic disorder is one of the main reasons for the growth retardation of gypsy moth larvae after feeding on the GM-colonized seedlings, while the improvement of gut environment and fat body metabolism in larvae results in the GI-induced insect susceptibility in *P. alba × P. berolinensis* seedlings. These findings may provide new insights for understanding how physiological alterations in herbivorous insects affect AMF-plant-herbivorous-insect interactions.

## Data Availability

All the data that support the findings of this study are available in the manuscript. The microbiota raw data have been deposited in the NCBI Sequence Read Archive (SRA, http://www.ncbi.nlm.nih.gov/sra, accessed on 8 May 2022) database, with the accession number PRJNA890777. The metabolome raw data have been deposited in the Metabolights (https://www.ebi.ac.uk/metabolights/, accessed on 8 May 2022) database, with the accession number MTBLS6182.

## Supplementary Materials

The following supporting information can be downloaded at: https://www.mdpi.com/article/10.3390/insects13111002/s1, Table S1 Root colonization rate by AMF in CK, GM and GI groups; Table S2 The top 30 genera in abundance; Table S3 The genera with significant changes in abundance between GM and CK groups; Table S4 The genera with significant changes in abundance between GI and CK groups; Table S5 Differentially accumulated metabolites in gypsy moth larvae of GM group; Table S6 Differentially accumulated metabolites in gypsy moth larvae of GI group; Figure S1 Venn diagram for operational taxonomic units in CK, GM and GI group; Figure S2 Linear discriminant analysis effect size (LEfSe) cladogram of gut microbiota in the Lymantria dispar larvae at the 5th instar after rearing on the leaves of GM or nonmycorrhizal-colonized plants; Figure S3 Linear discriminant analysis effect size (LEfSe) cladogram of gut microbiota in the Lymantria dispar larvae at the 5th instar after rearing on the leaves of GI or nonmycorrhizal-colonized plants; Figure S4 Summary of PICRUSt analysis between CK group and GM group or CK group and GI group.
