# Supplementary material for: Effects of Arbuscular Mycorrhizal Fungi-Colonized Populus alba × P. berolinensis Seedlings on the Microbial and Metabolic Status of Gypsy Moth Larvae
Source: Insects. 2022 Oct 31;13(11):1002. doi: 10.3390/insects13111002 (PMC9697668; doi:10.3390/insects13111002)
Supplement: Supplementary file 1 [file insects-13-01002-s001.zip › insects-1949475-supplementary.pdf]

Supplementary Materials

Table S1. Root colonization rate by AMF in CK, GM and GI groups.

	CK	GM	GI
30 d	0±0% c	10.0±0.5% b	15.6±0.8% a
60 d	0±0% c	20.1±1.3% b	30.5±2.1% a
90 d	0±0% c	45.6±2.5% b	61.7±3.2% a
100 d	0±0% c	43.7±2.6% b	60.8±3.0% a

Different lowercase letter indicates significant differences between control/treatment groups (N = 150; independent-samples *t*-test; $p < 0.05$).

Table S2. The top 30 genera in abundance.

Genus	CK1	CK2	CK3	CK4	GM1	GM2	GM3	GM4	GI1	GI2	GI3	GI4
g__*Pseudomonas*	58.42	59.40	68.50	63.66	65.25	68.53	48.87	58.31	9.75	9.47	9.84	10.66
g__Oxyphotobacteria_unclassified	1.50	2.22	1.92	1.87	4.02	6.32	9.53	4.67	71.88	73.37	75.56	73.78
g__*Ralstonia*	5.14	6.00	5.10	6.50	10.13	7.79	7.37	12.84	1.64	1.71	1.52	1.51
g__*Sphingopyxis*	1.81	1.96	1.41	3.24	4.23	3.98	10.70	5.55	2.70	3.91	3.15	3.37
g__*Brevundimonas*	1.99	2.07	2.90	2.95	3.19	1.95	4.87	4.05	1.76	1.65	1.63	0.80
g__*Staphylococcus*	4.89	3.84	4.46	3.56	1.56	0.68	1.28	0.00	0.00	0.11	0.00	0.49
g__Mitochondria_unclassified	0.21	0.12	0.11	0.02	0.09	0.28	0.21	0.11	3.39	3.41	2.65	4.68
g__*Rhizobacter*	0.51	1.38	0.73	0.47	0.92	0.77	1.99	1.55	1.14	1.30	1.58	1.05
g__TRA3-20_unclassified	0.47	0.29	0.11	0.38	1.28	1.57	1.56	0.88	0.63	0.34	0.32	0.44
g__*Methylibium*	0.23	0.44	0.41	0.57	0.47	0.86	2.06	1.05	0.59	0.42	0.70	0.30
g__*Acinetobacter*	0.03	0.05	0.07	0.00	1.86	0.13	0.08	4.11	0.08	0.01	0.00	0.08
g__*Caulobacter*	0.13	0.46	0.22	0.30	0.31	2.45	1.52	0.39	0.07	0.02	0.30	0.03
g__*Methylobacterium*	0.74	0.21	0.58	0.39	0.91	0.40	0.74	0.89	0.24	0.30	0.14	0.12
g__*Klebsiella*	2.55	0.91	0.41	0.46	0.14	0.10	0.19	0.00	0.00	0.13	0.00	0.02
g__*Streptococcus*	2.97	0.65	0.02	1.03	0.15	0.07	0.02	0.00	0.00	0.00	0.00	0.00
g__*Lactobacillus*	1.18	0.80	0.48	1.08	0.02	0.00	0.03	0.00	0.01	0.01	0.00	0.00
g__*Enterobacter*	0.34	0.82	0.89	0.89	0.05	0.11	0.14	0.00	0.00	0.00	0.00	0.05
g__Actinobacteria_unclassified	0.21	0.67	0.16	0.27	0.32	0.38	0.65	0.30	0.21	0.01	0.01	0.04
g__*Bradyrhizobium*	0.32	0.27	0.65	0.18	0.33	0.09	0.48	0.67	0.00	0.01	0.00	0.09
g__*Neisseria*	0.40	0.00	1.27	1.22	0.00	0.00	0.17	0.00	0.00	0.00	0.00	0.00
g__*Delftia*	0.58	0.26	0.09	0.29	0.70	0.17	0.12	0.73	0.05	0.00	0.01	0.05
g__*Dechloromonas*	0.13	0.26	0.33	0.23	0.13	0.44	0.61	0.00	0.08	0.22	0.17	0.14
g__*Corynebacterium*	0.08	0.48	0.00	1.01	0.00	0.00	0.06	0.00	0.22	0.41	0.00	0.17
g__*Ramlibacter*	0.18	0.24	0.11	0.08	0.15	0.24	0.75	0.28	0.11	0.02	0.12	0.07
g__Subgroup_7_unclassified	0.20	0.53	0.12	0.29	0.16	0.11	0.34	0.14	0.28	0.09	0.01	0.01
g__*Lutispora*	0.21	0.21	0.20	0.09	0.17	0.20	0.34	0.20	0.13	0.30	0.11	0.11
g__*Bacteroides*	0.70	0.51	0.33	0.02	0.00	0.02	0.08	0.30	0.32	0.00	0.00	0.00
g__*Methyloversatilis*	0.00	0.17	0.21	0.29	0.10	0.12	0.17	0.29	0.12	0.17	0.15	0.27
g__*Reyranella*	0.08	0.29	0.00	0.16	0.26	0.26	0.31	0.39	0.03	0.07	0.03	0.04
g__*Escherichia*	0.76	0.73	0.00	0.30	0.09	0.00	0.00	0.00	0.00	0.00	0.02	0.00

Table S3. The genera with significant changes in abundance between GM and CK groups.

Genus	CK1	CK2	CK3	CK4	GM1	GM2	GM3	GM4	Tendency
Subgroup_6_unclassified	0.02	0.00	0.00	0.00	0.18	0.35	0.14	0.26	Increased
Methylibium	0.23	0.44	0.41	0.57	0.47	0.86	2.06	1.05	Increased
Mesorhizobium	0.00	0.00	0.00	0.00	0.00	0.03	0.02	0.01	Increased
Thiobacillus	0.00	0.01	0.00	0.00	0.05	0.01	0.02	0.02	Increased
Oxyphotobacteria_unclassified	1.50	2.22	1.92	1.87	71.88	73.37	75.56	73.78	Increased
Mitochondria_unclassified	0.21	0.12	0.11	0.02	3.39	3.41	2.65	4.68	Increased
Subgroup_6_unclassified	0.02	0.00	0.00	0.00	0.19	0.10	0.27	0.02	Increased
Sphingosinicella	0.02	0.06	0.08	0.10	0.18	0.18	0.11	0.17	Decreased
Klebsiella	2.55	0.91	0.41	0.46	0.14	0.10	0.19	0.00	Decreased
Lactobacillus	1.18	0.80	0.48	1.08	0.02	0.00	0.03	0.00	Decreased
Lachnospiraceae_ND3007_group	0.02	0.00	0.00	0.01	0.00	0.00	0.00	0.00	Decreased
Ruminococcaceae_UCG-005	0.45	0.02	0.00	0.02	0.00	0.00	0.00	0.00	Decreased
Fusicatenibacter	0.03	0.08	0.03	0.04	0.00	0.00	0.00	0.00	Decreased
Akkermansia	0.01	0.02	0.00	0.01	0.00	0.00	0.00	0.00	Decreased
Ruminococcaceae_UCG-014	0.01	0.32	0.00	0.00	0.00	0.00	0.00	0.00	Decreased

Table S4. The genera with significant changes in abundance between GI and CK groups.

Genus	CK1	CK2	CK3	CK4	GI1	GI2	GI3	GI4	Tendency
Alphaproteobacteria_unclassified	0.00	0.00	0.00	0.00	0.01	0.01	0.00	0.00	Increased
Rosenbergiella	0.00	0.00	0.00	0.00	0.27	0.64	0.30	0.27	Increased
TRA3-20_unclassified	0.47	0.29	0.11	0.38	1.28	1.57	1.56	0.88	Increased
Acinetobacter	0.03	0.05	0.07	0.00	1.86	0.13	0.08	4.11	Increased
Ralstonia	5.14	6.00	5.10	6.50	10.13	7.79	7.37	12.84	Increased
Sphingopyxis	1.81	1.96	1.41	3.24	4.23	3.98	10.70	5.55	Increased
Oxyphotobacteria_unclassified	1.50	2.22	1.92	1.87	4.02	6.32	9.53	4.67	Increased
Staphylococcus	4.89	3.84	4.46	3.56	1.56	0.68	1.28	0.00	Increased
Chryseobacterium	0.07	0.00	0.15	0.00	0.00	0.00	0.00	0.00	Decreased
Ruminococcus]_torques_group	0.03	0.04	0.00	0.36	0.00	0.00	0.00	0.00	Decreased
Bradyrhizobium	0.32	0.27	0.65	0.18	0.00	0.01	0.00	0.09	Decreased
Bacteroides	0.70	0.51	0.33	0.02	0.32	0.00	0.00	0.00	Decreased
Bifidobacterium	0.84	0.07	0.00	0.01	0.00	0.00	0.00	0.00	Decreased
Actinobacteria_unclassified	0.21	0.67	0.16	0.27	0.21	0.01	0.01	0.04	Decreased
Delftia	0.58	0.26	0.09	0.29	0.05	0.00	0.01	0.05	Decreased
Alloprevotella	0.61	0.66	0.00	0.32	0.00	0.00	0.00	0.00	Decreased
Fusicatenibacter	0.03	0.08	0.03	0.04	0.00	0.00	0.00	0.00	Decreased
Fusobacterium	0.50	0.73	0.00	0.53	0.00	0.00	0.00	0.00	Decreased
Lachnospiraceae_ND3007_group	0.02	0.00	0.00	0.01	0.00	0.00	0.00	0.00	Decreased
Neisseria	0.40	0.00	1.27	1.22	0.00	0.00	0.00	0.00	Decreased
Brevundimonas	1.99	2.07	2.90	2.95	1.76	1.65	1.63	0.80	Decreased
Lactobacillus	1.18	0.80	0.48	1.08	0.01	0.01	0.00	0.00	Decreased
Klebsiella	2.55	0.91	0.41	0.46	0.00	0.13	0.00	0.02	Decreased
Streptococcus	2.97	0.65	0.02	1.03	0.00	0.00	0.00	0.00	Decreased
Ruminococcaceae_UCG-014	0.01	0.32	0.00	0.00	0.00	0.00	0.00	0.00	Decreased
Ralstonia	5.14	6.00	5.10	6.50	1.64	1.71	1.52	1.51	Decreased
Staphylococcus	4.89	3.84	4.46	3.56	0.00	0.11	0.00	0.49	Decreased
Pseudomonas	58.42	59.40	68.50	63.66	9.75	9.47	9.84	10.66	Decreased

Table S5. Differentially accumulated metabolites in gypsy moth larvae of GM group.

Metabolites	CK1	CK2	CK3	CK4	GM1	GM2	GM3	GM4	Tendency
Inosine 5'-monophosphate	23284	19924	20674	22646	29926	25342	27711	24352	Increased
L-propionylcarnitine	76895	95590	70313	104718	225862	186988	133299	167480	Increased
Phosphatidylcholine	65404	111507	102643	89211	224704	174342	177331	122610	Increased
2-Lysophosphatidylethanolamine	5435561	6809956	9450273	8437789	6798385	11455263	2162885	11597557	Increased
Phosphatidylethanolamine	29394	29146	23488	36886	35098	52230	56449	50454	Increased
Corticosterone	29913	45288	75915	47421	85351	61081	127726	100813	Increased
Acyl1-monogalactosyl-diacylglycerol	1014	655	1252	1881	1816	1661	2386	3124	Increased
Pantothenic acid	3096085	2521236	2745544	2960770	1065732	1463577	739893	1987166	Decreased
Uridine	16539	19522	19412	14993	8502	12404	9421	7075	Decreased
Cytarabine	8248	10084	8887	9066	7094	3504	4015	5017	Decreased
5,6-Dihydroxyindole-2-carboxylic acid	41036	44414	39573	9233	2039	1647	1117	760	Decreased
Isoleucine	1755686	1715466	1675548	1557700	1103660	1302762	1081689	1353734	Decreased
(-)-Epicatechin	95907	133320	228342	169743	56113	37002	46755	64579	Decreased
Penicillamine	824441	622032	915285	132190	63158	59150	36499	36547	Decreased
Pirbuterol	4031431	4428083	4014381	3525789	2994903	3349201	2061193	3014970	Decreased
Daidzin	22735	26784	28318	6061	1705	2738	9633	1123	Decreased
Hydroxykynurenine	62949	84512	56370	58474	49972	22707	19193	37018	Decreased
2-Furancarboxaldehyde	144099	151785	162730	98957	84627	100648	79338	92480	Decreased
Pyruvic acid	15934	14064	14952	6185	7498	5456	5808	5597	Decreased
L-kynurenine	147225	176975	166471	16048	4562	12627	48337	25117	Decreased
Alpha-ionone	21132	21939	19345	21641	13744	17142	1588	11695	Decreased
Linustatin	323890	323686	305036	123561	94433	153070	131452	146376	Decreased
N, N-dimethylformamide	83350	112296	111263	87857	66733	74151	79790	67055	Decreased
DL-ornithine	66046	69775	92873	60868	35887	44837	38524	37229	Decreased
5-Hydroxyindole-3-acetic acid	86686	142513	88387	18288	12339	21616	24241	24794	Decreased

Table S6. Differentially accumulated metabolites in gypsy moth larvae of GI group.

Metabolites	CK1	CK2	CK3	CK4	GI1	GI2	GI3	GI4	Tendency
Camptothecin	86686	142513	88387	18288	17822	14305	13388	18425	Increased
Phenylalanine	12603391	11636719	10339375	11516006	15214786	16182101	13594557	15812872	Increased
L-propionylcarnitine	76895	95590	70313	104718	163572	124579	206006	181693	Increased
9,10-Dhydroxy-12z-octadecenoic acid	3765	3504	4701	5138	5710	5703	5999	6554	Increased
Allantoic acid	1234173	1085477	1294752	1292400	1830156	1385115	1811091	1631709	Increased
Trans-cinnamic acid	124144	120632	109715	131272	180955	133365	182289	167381	Increased
(-)-Riboflavin	124566	103623	89819	102562	141158	138094	122089	163245	Increased
Arginine	991929	711925	666419	1169586	1369732	1219926	1751579	1478775	Increased
Salicylic acid	6549	7545	12082	5585	16550	13238	24376	16287	Increased
Indole	64147	48507	40147	36080	83364	58450	71686	73352	Increased
1H-indole-3-carboxaldehyde	339658	255004	237935	217838	345527	333719	359745	457948	Increased
Kynurenic acid	700760	585835	611751	635622	1462881	650902	1104851	1426245	Increased
L-tryptophan	3898180	2907708	2939775	2372635	3830584	3945688	3604323	4384417	Increased
3,4-Dihydroxy-L-phenylalanine	392742	565090	1150231	61485	1252674	1538899	855797	2074888	Increased
Pirbuterol	4031431	4428083	4014381	3525789	2494729	2987972	2653652	2453283	Decreased
Uridine	16539	19522	19412	14993	10740	8774	9748	3721	Decreased
Dodecanoic acid	7459	9076	8530	7210	5211	6326	4965	5953	Decreased
(-)-Epicatechin	95907	133320	228342	169743	12553	45709	17915	3954	Decreased
Pantothenic acid	3096085	2521236	2745544	2960770	1691642	2362522	1233272	1746143	Decreased
Isonicotinic acid	91764	96162	92549	85482	82882	73611	64087	73010	Decreased
Daidzin	22735	26784	28318	6061	4335	4578	2774	6314	Decreased
Pyridine	11569	11805	15132	14470	10541	8968	10268	10983	Decreased
N(6)-(1,2-dicarboxyethyl)AMP	40095	36584	30284	24765	21101	26482	17151	20363	Decreased
Beta-nicotinamide mononucleotide	25528	29935	34003	18771	4581	21123	7664	16221	Decreased
Trigonelline	1654149	1132421	1387384	1137389	787133	744551	941607	1121894	Decreased

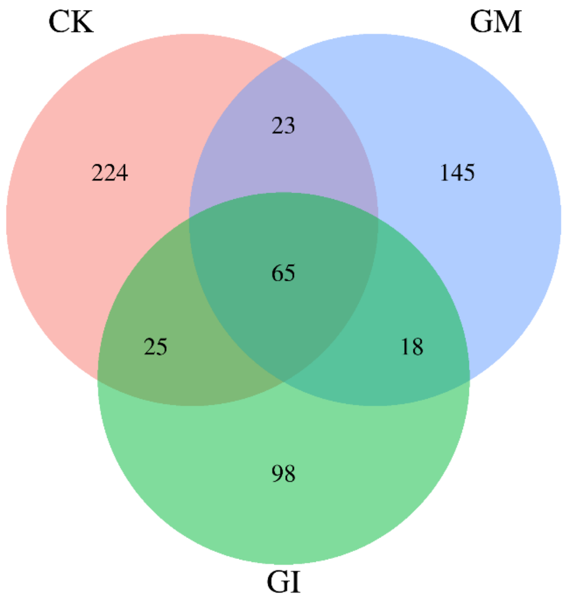

Figure S1. Venn diagram for operational taxonomic units in CK, GM and GI group.

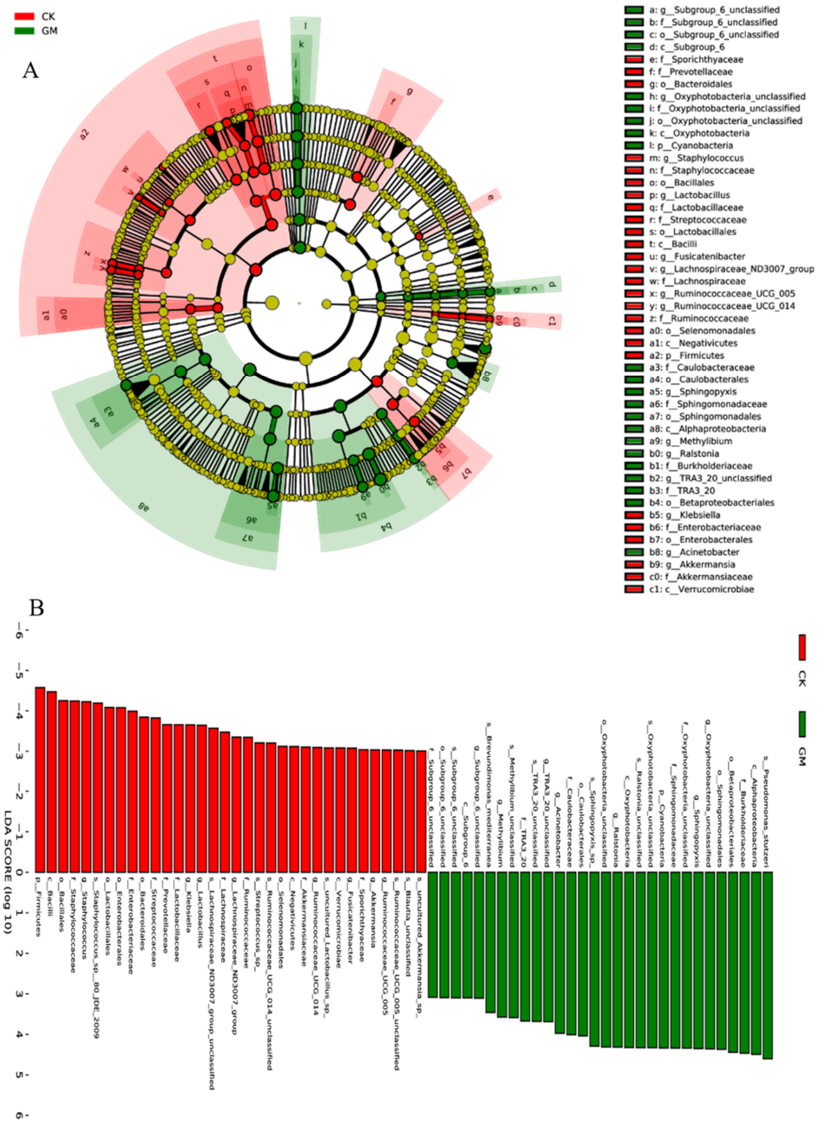

Figure S2. Linear discriminant analysis effect size (LEfSe) cladogram of gut microbiota in the *Lymantria dispar* larvae at the 5th instar after rearing on the leaves of GM or nonmycorrhizal-colonized plants. (**A**). Nodes that are not yellow mean a significant contribution to grouping. (**B**) LDA value of distribution histogram.

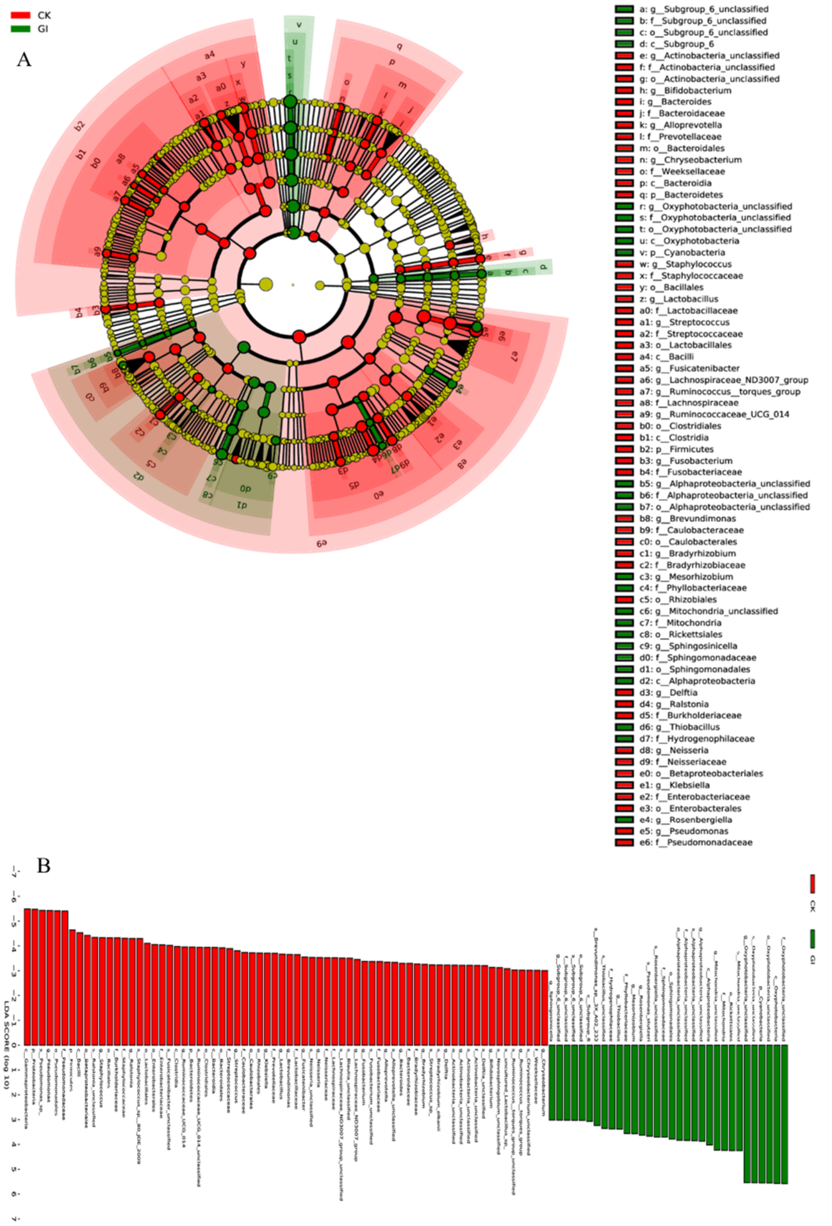

Figure S3. Linear discriminant analysis effect size (LEfSe) cladogram of gut microbiota in the *Lymantria dispar* larvae at the 5th instar after rearing on the leaves of GI or nonmycorrhizal-colonized plants. (**A**). Nodes that are not yellow mean a significant contribution to grouping. (**B**) LDA value of distribution histogram.

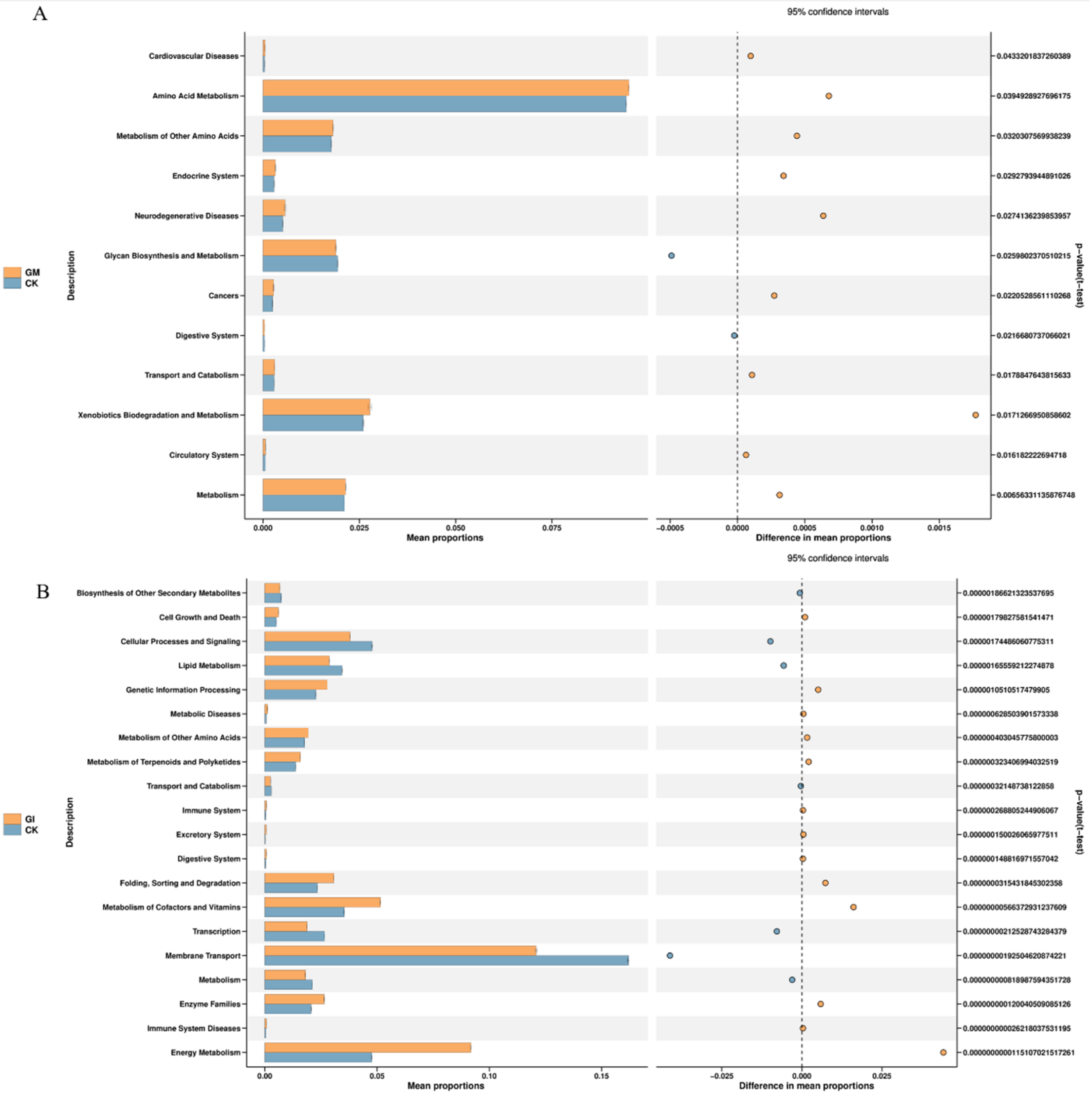

Figure S4. Summary of PICRUSt analysis between CK group and GM group (**A**) or CK group and GI group (**B**). Circles represent significant difference between CK group and GM/GI group (independent-samples t-test; $p < 0.05$).
